# Assessing and Mapping Reading and Writing Motivation in Third to Eight Graders: A Self-Determination Theory Perspective

**DOI:** 10.3389/fpsyg.2020.01678

**Published:** 2020-07-28

**Authors:** Fien De Smedt, Amélie Rogiers, Sofie Heirweg, Emmelien Merchie, Hilde Van Keer

**Affiliations:** ^1^Department of Educational Studies, Ghent University, Ghent, Belgium; ^2^Social Innovation Expertise Center, VIVES University of Applied Sciences, Kortrijk, Belgium; ^3^Preschool and Primary Education, Howest University of Applied Sciences, Bruges, Belgium

**Keywords:** reading motivation, writing motivation, self-determination theory, elementary education, secondary education

## Abstract

The twofold aim of this study was to substantiate the validity of the Self-Regulation Questionnaire-Reading Motivation and Self-Regulation Questionnaire-Writing Motivation for third to eight graders and to map motivational trends in elementary and secondary education students’ academic and recreational reading and writing. More specifically, we adopted the innovative and coherent theoretical framework of the Self-Determination Theory to study qualitatively different motives for reading and writing and to examine the relationships between them. In total, 2,343 students from third to eighth grade were involved. Based on confirmatory factor analyses, a two-factor model, distinguishing between autonomous and controlled motivation, for academic and recreational reading and writing was confirmed in all grades. Furthermore, the scales were reliable, and the measurement models were invariant across students’ gender and their general achievement level. Despite the absence of strong invariance for the measurement models across each of the different grades, we found evidence that students within the same grade level (i.e., middle elementary, upper elementary, and lower secondary grade) interpreted the SRQ-Reading and Writing scale items in a conceptually similar way. Factor correlations confirmed the interrelatedness of reading and writing motives, as well as strong associations between students’ motivation to read and write in either academic and recreational contexts. Finally, concerning the motivational trends, the present results advert to a significant decline of students’ autonomous motivation to read and write, both in and outside school. Accordingly, we point out that the late elementary and the lower secondary grades are crucial phases to engage students in motivating literacy activities. In light of these alarming results, we recommend future experimental research studies to focus on evaluating the effectiveness of instructional reading and writing activities that foster students’ innate need for autonomy, competence, and relatedness.

## Introduction

In November 2018, the European Literacy Network (ELN) signed a charter declaring engagement in perfecting and spreading literacy so that a truly human world, reflecting democratic values, can be established (European Literacy Network, 2018). Through this action, ELN aims to develop an integrated and inclusive approach to foster foundational literacy across Europe and to ensure that all its citizens have means to develop their literacy regardless of their individual background (e.g., with respect to language, age, socio-economic status, or disability). As such, ELN acknowledges that effective literacy skills, such as reading and writing, are crucial to participate in modern society. In this respect, Becker et al. (2010) state that the success of our knowledge society is dependent on the level of literacy of its population. Research, however, consistently points at causes for concern in this respect. As to reading performance, for example, large-scale international studies such as PIRLS and PISA documented that a substantial portion of students encounter difficulties with reading comprehension (Mullis et al., 2017; OECD, 2019). More specifically, PIRLS 2016 showed a significant decline in Flemish fourth grade students’ reading comprehension during the past decade (Mullis et al., 2017). Compared to the previous PISA results, the data of Flemish 15-year-old students in 2018 revealed similar worrying trends, namely a substantial decline in Flemish secondary education students’ reading performance (OECD, 2019). Similarly, writing assessment reports revealed alarming results on students’ poor writing performance (Inspectie van het Onderwijs, 2010; National Center for Education Statistics, 2012). In light of these findings, various empirical research studies refer to motivation as an important predictor of reading and writing performance (Graham et al., 2007; De Naeghel et al., 2012; Troia et al., 2013; De Smedt et al., 2016, 2018b; Teng and Zhang, 2018; Rocha et al., 2019; Rogiers et al., 2020; Toste et al., 2020), strongly emphasizing its key role in fostering both students’ reading and writing skills (Graham, 2018a, b). Notwithstanding the importance assigned to students’ motivation, reading motivation however appears to decline at the end of elementary education (Smith et al., 2012). Similarly, also writing motivation decreases as students progress through school (Cleary, 1991).

Given the above, motivational trends in reading and writing appear to evolve similarly, implying potential motivational reading–writing relationships. However, to study these reading–writing relations and to get more fine-grained insights into this downward trend of students’ reading and writing motivation across grades, comparable, reliable, and valid instruments are required. The present study, therefore, aims at (a) assessing students’ motives for reading and writing across elementary and secondary grades and (b) mapping motivational trends in students’ reading and writing. To gain in-depth understanding of these motives and of the relatedness between motives for reading and writing, an underlying, integrative motivation theory is essential. In this respect, several motivation theories have been adopted in reading and writing research, such as the self-efficacy theory (Bandura, 1977, 1997), expectancy-value theory (Wigfield and Eccles, 2000), achievement goal theory (Ames, 1992), and self-determination theory (SDT; Ryan and Deci, 2000a, 2020). In the present study, we adopt the innovative and coherent theoretical framework of SDT. We particularly opted for SDT because the theory not only focuses on the amount of students’ motivation (i.e., quantity of motivation) but also on the quality of motivation (i.e., the kind of motives underlying one’s behavior) (Ryan and Deci, 2000a, 2020). Studying students’ motivation to read and write within the SDT framework consequently enables us to not only study quantitative trends in students’ motivation throughout their school career, but also qualitative trends. Up to now, the SDT framework has only limitedly been adopted in reading and writing research. Especially studies investigating the relationships between reading and writing motivation, as conceptualized from an SDT perspective, are lacking. In what follows, we shortly review the literacy research field focusing on the interrelatedness between reading and writing and we highlight the current lack of motivational research within this research strand. Next, we present SDT as the underlying theoretical framework of the current study by elaborating on the value of this particular theory to examine reading and writing motivation in tandem. Finally, we provide an overview of research on assessing reading and writing motivation, particularly focusing on self-report measures based on SDT.

## Theoretical Background

### Reading and Writing Relations

Since the 70s of the 20th century, both reading and writing researchers provided considerable contributions to their respective research fields, mainly investigating reading and writing separately. Although reading and writing have cognitively different starting points (i.e., respectively receptive and productive by nature), they are closely related (Fitzgerald and Shanahan, 2000; Shanahan, 2016, 2019). Recently, the literacy research field in which the study of reading-writing connections is central, gained increased attention, both in theoretical and empirical research (Shanahan, 2016, 2019; Graham et al., 2018). In this respect, three theoretical models are especially relevant for research into the reading-writing nexus. Each framework describes particular ways in how reading and writing are connected (Shanahan, 2016, 2019; Graham et al., 2018). First, the *rhetorical relations theory*, which is socio-cognitive by nature, states that reading and writing are both communicative activities in which reader–writer relations and awareness are central. Empirical studies within this research strand have mainly focused on whether and how readers think about authors (i.e., author awareness) (e.g., Shanahan et al., 2011) and writers think about readers (i.e., audience awareness) (e.g., Lindgren et al., 2011). Second, the *functional theory* envisions reading and writing as functional activities that can be combined to accomplish specific learning goals. Functional investigations have studied the impact of combining reading and writing on two major outcomes, namely learning information from text (e.g., Merchie and Van Keer, 2016) and writing syntheses using multiple source texts (e.g., Mateos and Solé, 2009; Vandermeulen et al., 2020). Third, according to the *shared knowledge and process theory*, reading and writing depend on similar knowledge and cognitive processes. Empirical research that draws on this theory has focused on complex models to reveal patterns of relations between reading and writing (e.g., Shanahan and Lomax, 1986, 1988; Ahmed et al., 2014). To date, there is no consensus on the directionality of the models, with some studies suggesting unidirectional reading-to-writing (e.g., Ahmed et al., 2014) or writing-to-reading models (e.g., Berninger et al., 2002), while others provide evidence for bidirectional relationships between reading and writing (e.g., Abbott et al., 2010; Ahmed et al., 2014).

However, in the described theoretical models, it must be noticed that mainly cognitive and metacognitive variables have been studied so far. In this respect, reading and writing have mainly been studied from a cognitive perspective by primarily focusing on understanding how readers comprehend texts and on how writers compose texts (MacArthur and Graham, 2016). This is striking since the importance of motivational factors is increasingly highlighted in theoretical and empirical studies on both reading (e.g., De Naeghel et al., 2012) and writing (e.g., De Smedt et al., 2018b). Consequently, the relations between students’ motives to engage in reading and in writing is an underexplored area within the current literacy research field. To study the interrelatedness of these motives, an unequivocal motivation theory is essential in the light of conceptualizing and measuring the key motivational factors.

### Reading and Writing Motivation Within the Self-Determination Theory

Self-Determination Theory (SDT) is a promising, contemporary motivation theory with a continuously emerging empirical base in educational contexts (e.g., Mouratidis et al., 2018; Aelterman et al., 2019). Over the last decade, interest in SDT increased substantially in the field of language learning (e.g., Guay et al., 2010; De Naeghel et al., 2012; De Smedt et al., 2018a, 2020; Rocha et al., 2019). This theory posits that students do not only vary in the quantity but also in the quality of their motivation (i.e., the kind of motives underlying one’s behavior) (Ryan and Deci, 2000a, 2020). Based on this assumption, SDT describes a full continuum of motivation and differentiates between qualitatively different types of motivation: (a) amotivation (e.g., lack of motivation to read or write), (b) external regulation (e.g., reading or writing because of experiences of external pressure, such as obtaining a reward), (c) introjected regulation (e.g., reading or writing because of experiences of internal pressure, such as shame), (d) identified regulation (e.g., reading or writing because of personally valuing the activities), and (e) intrinsic regulation (e.g., reading or writing because of an inherently enjoyment of the activities). Within SDT-research in general and in the field of language learning in particular (Ryan and Deci, 2000a, 2020; De Naeghel et al., 2012; De Smedt et al., 2018b), the last four types coincide two by two in respectively controlled and autonomous motivation. Autonomous motivation is the most optimal type of motivation and refers to engaging in an activity with an inner sense of satisfaction or because one values the activity (i.e., intrinsic and identified regulation; Ryan and Deci, 2000a, 2020). Contrary to autonomous motivation, controlled motivation refers to engaging in an activity because of internal or external pressure (i.e., external and introjected regulation; Ryan and Deci, 2000a, 2020). In the current study, we did not consider the concept of amotivation further, as we mainly focused on qualitatively different reasons that students display for reading and writing rather than on the degree to which they are motivated.

Until now, SDT has rarely been adopted in reading and writing research. The limited number of SDT studies in reading and writing in upper-elementary grades (cf., 11–12-year-old students), however, revealed promising insights into the advantages of distinguishing autonomous and controlled motivation. For instance, De Naeghel et al. (2012) showed that autonomous reading motivation is related to more positive reading behavior and improved reading comprehension performance. As to writing, De Smedt et al. (2016) found that autonomously motivated students write qualitatively better texts while controlled motivated students were significantly less successful in their writing. Based on these empirical findings indicating positive relationships between autonomous motivation and reading and writing outcomes on the one hand and negative relations between controlled motivation and reading and writing performance on the other hand, the need to map these motives throughout students’ academic career becomes apparent. By gaining in-depth insights into these qualitatively different types of reading and writing motivation (i.e., autonomous and controlled motivation) and by studying motivational trends as students progress through school, we will gain a deeper understanding in how we can foster the most optimal motives for reading and writing throughout students’ academic career. In this respect, SDT points to the importance of psychological needs satisfaction in learning environments in order to foster students’ autonomous motivation. More specifically, three basic needs are seen as particularly fundamental. First, the need for autonomy refers to a sense of initiative and ownership in one’s actions. Second, the need for competence entails feelings of mastery and a sense that one can succeed. Finally, the need for relatedness refers to a sense of belonging and being related to significant others. To nurture these innate psychological needs, teachers can adopt a need-supportive teaching style characterized by autonomy-supportive, structured, and involved teacher behavior (Soenens and Vansteenkiste, 2005). Blocking of any of these three basic needs is seen as damaging for students’ autonomous motivation (Ryan and Deci, 2000b, 2020).

### Assessing Reading and Writing Motivation

As self-report questionnaires are feasible instruments to examine large sample sizes, students’ reading and writing motivation are traditionally assessed by means of such questionnaires (e.g., Wigfield and Guthrie, 1997; De Naeghel et al., 2012; Troia et al., 2013; De Smedt et al., 2018b; Rocha et al., 2019). In this respect, prior research draws particular attention to the following insights in view of assessing students’ reading and writing motivation in a reliable and valid manner. First, students’ reading and writing experiences (e.g., learning different literacy skills and strategies or experiencing diverse educational reading and writing practices) may influence how students respond to these self-report questionnaires. In this respect, Hamilton et al. (2013) stressed that reading and writing motivation scales should be sensitive to students’ reasons for learning to read and write. Second, different motivational dynamics might occur in recreational and academic literacy contexts. For instance, students might have different reasons or motives to read or write for school compared to leisure-time reading and writing (De Naeghel et al., 2012). Third, Wigfield (1997) underlined the need to study motivation in a domain-specific way. In this respect, motivation scales should assess reading and writing motivation domain-specifically rather than generally across domains and school subjects (e.g., motivation for literacy) (Guay et al., 2010). Finally, students may experience reading and writing differently throughout their school career, indicating possible grade-level differences in how students interpret items on self-report questionnaires (Hamilton et al., 2013). In this respect, Hamilton et al. (2013) reported reliable and valid domain-specific measures for reading and writing within the theoretical framework of the achievement goal theory (Ames, 1992). More specifically, the reading items loaded consistently across grades (i.e., grade 2 through 7), while the interpretation of the writing items differed for younger and older students.

Within the limited amount of SDT research on reading and writing motivation, some studies focused on the development of domain-specific self-report measures (Guay et al., 2010; De Naeghel et al., 2012; De Smedt et al., 2018b; Rocha et al., 2019; Limpo et al., 2020). For instance, De Naeghel et al. (2012), developed and validated the Self-Regulation Questionnaire-Reading Motivation (SRQ-Reading Motivation), which is a reliable and valid questionnaire to measure fifth and sixth-graders’ reading motivation in both academic and recreational contexts. More specifically, factor analyses showed that the two-factor model (i.e., distinguishing autonomous and controlled motivation) was the best fit to the data compared to the four-factor model (i.e., distinguishing intrinsic, identified, introjected, and extrinsic regulation). Furthermore, the measurement model was invariant across gender, indicating that boys and girls interpreted the items similarly. To date, this questionnaire has not been tested and validated in middle elementary grades and has only recently been adopted in lower secondary grades (Van Ammel et al., unpublished). Based on the work of De Naeghel et al. (2012), De Smedt et al. (2018b) developed and tested the Self-Regulation Questionnaire-Writing Motivation (SRQ-Writing Motivation) in upper elementary education (i.e., grades 5 and 6). This questionnaire contains the same set of items as the SRQ-Reading Motivation, but the initial reading-oriented items were translated to the context of writing. De Smedt et al. (2018b), however, only addressed writing motivation in an academic context. Factor analyses confirmed the two-factor model of the SRQ-Writing Motivation (i.e., distinguishing autonomous and controlled motivation). Furthermore, invariance tests showed that the measurement model was invariant across gender and general achievement level. To our knowledge, this questionnaire has not been tested in middle elementary grades, nor in lower secondary grades.

### Aim of the Present Study

To date, there is little research on assessing and mapping reading and writing motivation departing from the framework of SDT. Furthermore, within the limited research available, different theoretical conceptualizations and instruments within different educational contexts are used. To map and relate students’ motives for reading and writing and to investigate motivational trends throughout students’ school careers, comparable, valid, and reliable assessment methods are needed. Therefore, the present study investigates whether previously developed SDT-based domain-specific reading (De Naeghel et al., 2012) and writing motivation instruments (De Smedt et al., 2018b) can be used across different educational grades. This study extends prior research by (a) assessing qualitatively different types of both reading and writing motivation within the same study, (b) studying these types of motivation in the academic as well as in the recreational context, (c) investigating reading and writing motivation in both elementary and secondary education, (d) studying measurement invariance according to different student characteristics (i.e., gender, achievement level, and grade), and (e) investigating the interrelatedness between reading and writing motivation. The following research questions (RQ) are central in this study:

• RQ1: Can the previously developed SRQ-Reading Motivation and SRQ-Writing Motivation scales be used across gender, general achievement, and educational grade?
• RQ2: What are the relationships between reading and writing motivation?
• RQ3: What are the trends in reading and writing motivation in both the academic and recreational context throughout elementary and secondary education?

Based on previous empirical and theoretical research, we put forward the following hypotheses: First, departing from the theoretical framework of the achievement goal theory (Ames, 1992), Hamilton et al. (2013) reported reliable and valid domain-specific measures for reading and writing. More specifically, they found that the reading items loaded consistently across grades (i.e., grade 2 through 7), while the interpretation of the writing items differed for younger and older students. Based on the study of Hamilton et al. (2013), we hypothesize possible grade-level differences in the SRQ-Writing Motivation items, which draw upon SDT (Ryan and Deci, 2000a, 2020). Second, based on theoretical and empirical research on reading-writing connections (Shanahan, 2016, 2019; Graham et al., 2018) and on previous studies regarding reading and writing motivation in Flanders (De Naeghel et al., 2012; De Smedt et al., 2018b), we anticipate high correlations between students’ autonomous reading and writing motivation on the one hand, and their controlled reading and writing motivation on the other hand. Finally, given the general decline in academic intrinsic motivation throughout students’ school careers (Gottfried et al., 2001; Bouffard et al., 2003; Corpus et al., 2009; Gnambs and Hanfstingl, 2016) and based on the reported significant decline in Flemish students’ reading performance the past decade (Mullis et al., 2017; OECD, 2019), we hypothesize a significant decline in Flemish students’ reading and writing motivation as it relates to higher educational grades.

## Materials and Methods

### Educational Context

Elementary education in Flanders (Belgium) is organized for children from 6 to 12 years old and comprises six subsequent school years (i.e., first to sixth grade). Secondary education is intended for adolescents from 12 to 18 and contains three stages of two grades (i.e., seventh to twelfth grade). Students in the lower-secondary grades (i.e., seventh and eighth grade) are offered a common curriculum. At the start of ninth grade, students make a choice of study and from that grade onward, four different types of education are offered (i.e., general, technical, vocational, and arts education). The Flemish government ensures the quality of its education by imposing attainment targets which are minimum objectives found necessary and attainable for students. The attainment targets for elementary students on the one hand and for secondary students on the other hand explicitly reflect upon knowledge, skills, and attitudes. In light of reading and writing attitudes, the attainment targets more specifically state that students should develop attitudes such as reading and writing readiness and pleasure (Flemish Ministry of Education and Training, 2005).

### Participants

In total, 2,343 students from 127 classes from 26 different elementary and secondary schools in Flanders (Belgium) were involved in this study. More specifically, 440 third (18.8%), 445 fourth (18.9%), 452 fifth (19.3%), 391 sixth (16.7%), 283 seventh (12.1%), and 332 eighth graders (14.2%) participated. Almost half of the participating students were female (49.2%) and the majority of the students spoke Dutch (i.e., the language of instruction in Flanders) as their home language (84.3%), while 10.3% were bilingual and only 5.4% spoke a foreign home language. Since experienced teachers are believed to make accurate judgments on students’ general achievement level (Südkamp et al., 2012), the teachers of the participating students were asked to evaluate students’ general achievement level (i.e., general performance across subjects) by typifying each student as (a) a below average or low achiever, (b) an average achiever, or (c) an above average or high achiever. As to students’ general achievement level, respectively 21.5, 53.8, and 24.7% of the students were considered low, average, and high achievers. Based on the statistical overview of the Flemish education in 2016–2017 (i.e., academic year in which the data was collected; Flemish Ministry of Education and Training, 2017) and based on previous large-scale studies in Flemish elementary and secondary schools (De Smedt et al., 2018b; Rogiers et al., 2020), the student samples in this study are representative for the elementary and secondary school population in Flanders. Table 1 summarizes students’ characteristics per grade level.

**TABLE 1:** Overview of student characteristics in middle and upper elementary grades and in lower secondary grades.

	Middle elementary grades^a^		Upper elementary grades^b^		Lower secondary grades^c^		All students	
	*M*	*SD*	*M*	*SD*	*M*	*SD*	*M*	*SD*
Age (years)	9.23	0.67	11.21	0.65	13.26	0.69	10.94	1.70

	***N***	**%**	***N***	**%**	***N***	**%**	***N***	**%**

**Gender**								
Male	425	48.02	421	49.94	307	49.91	1153	49.21
Female	460	51.98	422	50.06	308	50.09	1190	50.79
Total	885	100.00	843	100.00	615	100.00	2343	100.00
**Home language**								
Dutch	722	81.86	685	81.45	565	91.87	1972	84.35
Other language	47	5.33	37	4.40	42	6.83	126	5.39
Dutch + other language	113	12.81	119	14.15	8	1.30	240	10.26
Total	882	100.00	841	100.00	615	100.00	2338	100.00
**Grade**								
Third grade	440	49.72	0	0.00	0	0.00	440	18.77
Fourth grade	445	50.28	0	0.00	0	0.00	445	18.99
Fifth grade	0	0.00	452	53.62	0	0.00	452	19.29
Sixth grade	0	0.00	391	46.38	0	0.00	391	16.69
Seventh grade	0	0.00	0	0.00	283	45.85	283	12.08
Eight grade	0	0.00	0	0.00	332	54.15	332	14.18
Total	885	100.00	843	100.00	615	100.00	2343	100.00
**Achievement level**								
Low	209	24.27	175	20.76	79	17.75	463	21.54
Average	443	51.45	493	58.48	220	49.43	1156	53.79
High	209	24.28	175	20.76	146	32.82	530	24.67
Total	861	100.00	843	100.00	445	100.00	2149	100.00

*^a^Third and fourth grade. ^b^Fifth and sixth grade. ^c^Seventh and eighth grade.*

### Procedure

The data were collected by means of student questionnaires. The data collection procedure, however, was slightly different in elementary and secondary grades. As to the elementary grades, 61 undergraduate students administered student questionnaires during regular class hours while the teacher was present in the class. The undergraduate students were trained in administering the questionnaires following a strict administration protocol. According to this protocol, the participants were first provided with directions and practice items to prepare them for the actual questionnaire. Afterward, they completed the questionnaires individually, while the undergraduate students were present to clarify items that were not clear. As to administration in secondary education, the participants completed an online version of the questionnaires during regular class periods in the presence of their teacher and a trained undergraduate student.

### Measures

Students completed the questionnaires containing the following sections: First, students provided background information concerning their gender, home language, and date of birth. Second, they completed the SRQ-reading motivation (De Naeghel et al., 2012) for both academic and recreational reading. Third, students completed the SRQ-academic writing motivation (De Smedt et al., 2018b). Finally, students were asked whether they write texts in their free time (0 = no, 1 = yes). Students responding positively were forwarded to the questionnaire on recreational writing. As both the SRQ-Reading Motivation and SRQ-Writing Motivation have been developed and administered in Flanders in prior studies, the Dutch items were at our disposal (De Naeghel et al., 2012; De Smedt et al., 2018b).

#### Reading Motivation

Students’ reading motivation was measured using the SRQ-Reading motivation (De Naeghel et al., 2012), which consists of 17 items to be scored on a five-point Likert scale ranging from “disagree a lot” to “agree a lot.” The questionnaire contains each item twice for measuring reading in two contexts: one for measuring academic reading motivation (e.g., “I read for school, because I like reading”) and one for measuring recreational reading motivation (e.g., “I read in my free time, because I like reading”). Factor analyses in the study of De Naeghel et al. (2012) revealed that the SRQ-Reading Motivation measures two types of academic and recreational reading motivation: autonomous (e.g., “I read for school because I think reading is meaningful”; “I read in my free time because I really like it”) and controlled reading motivation (e.g., “I read for school because I will feel guilty if I don’t do it”; “I read in my free time because others will punish me if I don’t read”).

#### Writing Motivation

To assess students’ writing motivation, the SRQ-Writing motivation was administered (De Smedt et al., 2018b). This questionnaire contains the same set of 17 items as the SRQ-Reading Motivation, but the initial reading-oriented items were translated to writing contexts. Factor analyses in the study of De Smedt et al. (2018b) revealed that the SRQ-Writing Motivation distinguishes two types of academic writing motivation as well: autonomous (e.g., “I write for school because it is important to me to write”; “I write for school because I enjoy writing”) and controlled reading motivation (e.g., “I write for school because I will feel ashamed of myself if I don’t write”; “I write for school because others will only reward me if I write”). In the present study, autonomous and controlled writing motivation was also assessed for two different contexts (i.e., academic writing and recreational writing).

### Data Analysis

#### Confirmatory Factor Analyses and Reliability Analyses

To examine the structure and the reliability (RQ1) of the SRQ-Reading and Writing Motivation, confirmatory factor analyses (CFA) were conducted and estimators of internal consistency (Bentler, 2009) were calculated. To evaluate the model fit, the following robust fit indices are reported: (a) the chi-square test statistic and *p*-value, (b) the comparative fit index (CFI), (c) the root mean square error of approximation (RMSEA), and (d) the standardized root mean residual (SRMR). Browne and Cudeck (1992) stated that CFI should be above 0.90 for adequate fit. Furthermore, a cut-off value for RMSEA close to 0.06 is necessary for an acceptable fit (Hu and Bentler, 1999), while a value lower than 0.08 indicates a reasonable fit (Schreiber et al., 2006). The value of SRMR of 0.08 or lower indicates acceptable fit (Hu and Bentler, 1999). For the CFA we used RStudio 3.6.1. (R Core Team, 2019), lavaan package 0.6-5 (Rosseel, 2012). We additionally used the semTools 0.5-2 package to calculate the internal consistencies of the scales (Jorgensen et al., 2019).

#### Multiple Group Measurement Invariance

We conducted multiple group measurement invariance (MG-MI) to test whether the factor structure of the instruments is invariant across gender (i.e., boys and girls), general achievement level (i.e., low, average, and high achievers), and grades (third, fourth, fifth, sixth, seventh, and eighth grade) (RQ 1). To examine MG-MI, we tested a sequence of nested models with varying equality constraints. The baseline model was tested for equivalent factor structures (i.e., configural invariance). The subsequent models tested more conservative restrictions, more specifically weak invariance (i.e., equal loadings) and strong invariance (i.e., equal loadings and intercepts). Differences between nested models were determined by means of changes in CFI. According to Cheung and Rensvold (2002), ΔCFI should be smaller than or equal to 0.01. For the MG-MI analyses we used RStudio 3.6.1. (R Core Team, 2019), lavaan package 0.6-5 (Rosseel, 2012).

#### Non-normal and Clustered Data

Because the data were not normally distributed (skewness values ranging from −1.014 to 0.664 and kurtosis values ranging from −0.739 to 1.137), we applied the robust maximum likelihood estimation method with a Yuan Bentler (YB) scaled chi-square test statistic in CFA and MG-MI (Chou et al., 1991; Yuan and Bentler, 2000). Furthermore, we adjusted the standard errors and fit statistics by taking the clustered nature (i.e., students nested in classes) of the data into account (Muthén and Satorra, 1995; Stapleton, 2006).

#### Correlational Analyses

Once the final structure of the scales and reliabilities were determined, correlational analyses were used to study the relationships between the reading and writing motivation scales (RQ2). More specifically, factor correlations were examined using RStudio 3.6.1. (R Core Team, 2019), lavaan package 0.6-5 (Rosseel, 2012).

#### Descriptive and Multilevel Analyses

Based on the CFA and MG-MI, we used the final scale scores to conduct descriptive analyses. The descriptive analyses were used to investigate motivational trends in reading and writing (RQ3). Furthermore, the differences between the different grades of a grade level were tested using MLwiN 2.32, thereby taking the clustered nature of the data into account (Rasbash et al., 2012).

## Results

All 2,343 students completed the SRQ-Reading Motivation for academic and recreational reading and the SRQ-Writing Motivation for academic writing. 1,020 students (43.5%) indicated that they write during their free time and subsequently completed the SRQ-Writing Motivation for recreational writing.

### RQ1: The Structure and Reliability of the Scales and Measurement Invariance

As to the first research question, we managed to substantiate the validity of both the SRQ-Reading Motivation and SRQ-Writing Motivation. Parallel with the work of De Naeghel et al. (2012) and De Smedt et al. (2018b), a correlation between the error terms of two autonomous motivation items on the one hand and between two controlled motivation items on the other hand was allowed. More specifically, respectively between the autonomous motivation items “I read/write a text because I really like it” and “I read/write a text because it’s fun to read/write” and between the controlled motivation items “I read/write a text because I have to prove to myself that I can get good reading/writing grades” and “I read/write a text because I can just be proud of myself if I get good reading/writing grades.” First, we explored an initial measurement model that included the SRQ-Reading and Writing Motivation data in the academic and recreational context. Given the non-satisfactory fit statistics [YBχ^2^_(__2174__)_ = 14242.749, *p* < 0.001, CFI = 0.840, RMSEA = 0.049, SRMR = 0.078], we proceeded with the analysis of four separate measurement models: (a) academic reading motivation, (b) recreational reading motivation, (c) academic writing motivation, and (d) recreational writing motivation. Based on CFA of the SRQ-Reading and Writing Motivation data, the fit of the expected two-factor model was good in both academic and recreational contexts (see Table 2). More specifically, CFI values were above 0.90 (i.e., values ranging from 0.918 to 0.956), RMSEA values were close to 0.06 (i.e., values ranging from 0.055 to 0.080), and SRMR values were lower than 0.08 (i.e., values ranging from 0.058 to 0.064) indicating an acceptable fit. The items of the SRQ-Reading and Writing Motivation are presented in Table 3, along with standardized factor loadings for these items. Factor loadings for autonomous reading and writing motivation were acceptable and ranged from 0.47 to 0.89. The factor loadings of the majority of the controlled reading and writing motivation items were acceptable (i.e., ranging from 0.59 to 0.78), except for the items regarding motives for reading and writing in terms of getting good grades (i.e., factor loadings ranging from 0.36 to 0.49). Furthermore, reliability analyses revealed good internal consistencies ranging from Bentler’s ρ = 0.80 to Bentler’s ρ = 0.94 (see Table 4). We refer readers interested in the structure and reliability of the SRQ-Reading and Writing Motivation scales in middle and upper elementary, and/or lower secondary education to the Supplementary Tables. More specifically, the fit statistics per grade level are presented in Supplementary Table 1, the standardized factor loadings of the scales for each grade level are presented in Supplementary Tables 2–5, and the reliability measures per grade level can be found in Supplementary Table 6.

**TABLE 2:** Confirmatory factor analyses on the SRQ-reading and writing motivation: summary of goodness-of-fit statistics.

	YBχ^2^	*df*	CFI	RMSEA	SRMR
Academic Reading Motivation	772.715***	116	0.956	0.055	0.058
Recreational Reading Motivation	1127.497***	116	0.943	0.073	0.064
Academic Writing Motivation	1147.748***	116	0.937	0.071	0.071
Recreational Writing Motivation	687.707***	116	0.918	0.080	0.064

***p < 0.001, **p < 0.01, *p < 0.05.

**TABLE 3:** SRQ-reading and writing motivation: items and standardized factor loadings for academic and recreational reading and writing.

Item	Autonomous		Controlled		*R*^2^	
**^a^I read for school because…**	AC^a^	RE^b^	AC	RE	AC	RE
**^b^I read in my free time because…**						
I **enjoy** reading.	0.77	0.86			0.60	0.73
I think it is **very useful** for me to read.	0.77	0.82			0.60	0.67
It’s **fun** to read.	0.85	0.88			0.73	0.77
I **really like it**.	0.85	0.87			0.72	0.76
I think reading is **meaningful**.	0.76	0.83			0.58	0.68
I think reading is **interesting**.	0.85	0.89			0.72	0.78
It is **important to me to read**.	0.74	0.78			0.55	0.61
I think reading is **fascinating**.	0.63	0.66			0.39	0.43
I don’t want to **disappoint others**.			0.61	0.74	0.37	0.55
That is what **others expect me to do**.			0.61	0.74	0.37	0.55
I will feel **guilty** if I don’t do it.			0.67	0.73	0.45	0.53
**Others will only reward me if I read**.			0.59	0.66	0.35	0.44
I have t**o prove to myself that I can get good reading grades**.			0.40	0.49	0.16	0.24
**Others will punish me** if I don’t read.			0.59	0.60	0.35	0.36
I will feel **ashamed** of myself if I don’t read.			0.68	0.71	0.47	0.50
**Others think that I have to**.			0.65	0.69	0.43	0.47
I can just be **proud of myself if I get good reading grades**.			0.36	0.46	0.13	0.21
**^a^I write a text for school because…**						
**^b^I write a text in my free time because…**						
I **enjoy** writing.	0.80	0.74			0.63	0.55
I think it is **very useful** for me to write.	0.76	0.69			0.58	0.48
It’s **fun** to write.	0.84	0.81			0.71	0.65
I **really like it**.	0.83	0.74			0.70	0.55
I think writing is **meaningful**.	0.79	0.69			0.63	0.47
I think writing is **interesting**.	0.85	0.79			0.73	0.63
It is **important to me to write**.	0.76	0.66			0.58	0.44
I think writing is **fascinating**.	0.67	0.47			0.45	0.22
I don’t want to **disappoint others**.			0.71	0.77	0.51	0.59
That is what **others expect me to do**.			0.69	0.78	0.47	0.61
I will feel **guilty** if I don’t do it.			0.73	0.78	0.53	0.61
**Others will only reward me if I write**.			0.59	0.70	0.34	0.49
I have t**o prove to myself that I can get good writing grades**.			0.39	0.44	0.15	0.20
**Others will punish me** if I don’t write.			0.60	0.66	0.36	0.43
I will feel **ashamed** of myself if I don’t write.			0.70	0.77	0.48	0.60
**Others think that I have to**.			0.71	0.77	0.51	0.59
I can just be **proud of myself if I get good writing grades**.			0.37	0.45	0.14	0.21

*^a^Academic context. ^b^Recreational context.*

**TABLE 4:** Reliability measures: SRQ-reading and writing motivation.

	Bentler’s ρ
**Academic reading motivation**	
Autonomous	0.92
Controlled	0.80
**Recreational reading motivation**	
Autonomous	0.94
Controlled	0.83
**Academic writing motivation**	
Autonomous	0.93
Controlled	0.83
**Recreational writing motivation**	
Autonomous	0.87
Controlled	0.85

Furthermore, we studied MG-MI across gender, general achievement level, and grades. Tables 5, 6 present a summary of goodness-of-fit statistics for the SRQ-Reading Motivation and SRQ-Writing Motivation, respectively. Small changes in the CFI and satisfying overall model results revealed strong invariance for the measurement models across gender and general achievement level (ΔCFI values ranging from 0.000 to 0.006). It was, however, not possible to confirm strong invariance for the measurement models across the different grades (ΔCFI values ranging from 0.015 to 0.026) indicating that third, fourth, fifth, sixth, seventh, and eighth graders interpreted the SRQ-Reading and Writing Motivation items differently. Based on this finding, we additionally investigated whether students within the same grade level (i.e., middle elementary: third and fourth grade; upper elementary: fifth and sixth grade, and lower secondary grade: seventh and eighth grade) interpreted the items in a conceptually similar way. Here, the results showed strong invariance for the measurement models across grades within the same grade level (ΔCFI values ranging from 0.000 to 0.006), indicating that respectively (a) third and fourth graders, (b) fifth and sixth graders, and (c) seventh and eighth graders interpreted the items similarly (see Tables 7, 8).

**TABLE 5:** Multiple-group measurement invariance testing on SRQ-academic and recreational reading motivation across gender, general achievement level, and grades: summary of goodness-of-fit statistics.

Measurement invariance tests	Overall results						Compared models	Model difference results					
	YBχ^2^	*df*	*p*	CFI	RMSEA	SRMR		ΔYBχ^2^	Δ*df*	*p*	ΔCFI	ΔRMSEA	ΔSRMR
**Academic reading – Gender**													
Configural invariance	917.823	232	0.000	0.954	0.056	0.061							
Weak invariance	944.794	247	0.000	0.953	0.055	0.061	Model 1 vs. model 2	26.971	15	0.028	0.001	0.001	0.000
Strong invariance	1003.495	262	0.000	0.950	0.055	0.062	Model 2 vs. model 3	58.701	15	0.000	0.003	0.000	0.001
**Academic reading – General achievement**													
Configural invariance	973.523	348	0.000	0.953	0.051	0.062							
Weak invariance	1032.365	378	0.000	0.952	0.050	0.065	Model 1 vs. model 2	58.842	30	0.001	0.001	0.001	0.003
Strong invariance	1106.285	408	0.000	0.949	0.054	0.066	Model 2 vs. model 3	73.920	30	0.000	0.003	0.004	0.001
**Academic reading – Grade**													
Configural invariance	1424.924	696	0.000	0.955	0.055	0.060							
Weak invariance	1622.065	771	0.000	0.949	0.057	0.071	Model 1 vs. model 2	197.141	75	0.000	0.006	0.002	0.011
Strong invariance	2118.535	846	0.000	0.923	0.066	0.080	Model 2 vs. model 3	496.470	75	0.000	0.026	0.011	0.009
**Recreational reading – Gender**													
Configural invariance	1234.284	232	0.000	0.943	0.072	0.065							
Weak invariance	1285.866	247	0.000	0.942	0.071	0.068	Model 1 vs. model 2	51.582	15	0.000	0.001	0.001	0.003
Strong invariance	1338.127	262	0.000	0.938	0.070	0.068	Model 2 vs. model 3	52.261	15	0.000	0.004	0.001	0.000
**Recreational reading – General achievement**													
Configural invariance	1318.664	348	0.000	0.941	0.073	0.067							
Weak invariance	1361.648	378	0.000	0.941	0.070	0.068	Model 1 vs. model 2	42.984	30	0.059	0.000	0.003	0.001
Strong invariance	1458.539	408	0.000	0.937	0.069	0.070	Model 2 vs. model 3	96.891	30	0.000	0.004	0.001	0.002
**Recreational reading – Grade**													
Configural invariance	1828.522	696	0.000	0.940	0.073	0.067							
Weak invariance	1985.664	771	0.000	0.936	0.072	0.077	Model 1 vs. model 2	157.142	75	0.000	0.004	0.001	0.010
Strong invariance	2429.034	846	0.000	0.918	0.078	0.087	Model 2 vs. model 3	443.370	75	0.000	0.018	0.006	0.010

**TABLE 6:** Multiple-group measurement invariance testing on SRQ-academic and recreational writing motivation across gender, general achievement level, and grades: summary of goodness-of-fit statistics.

Measurement invariance tests	Overall results						Compared models	Model difference results					
	YBχ^2^	*df*	*p*	CFI	RMSEA	SRMR		ΔYBχ^2^	Δ*df*	*p*	ΔCFI	ΔRMSEA	ΔSRMR
**Academic writing – Gender**													
Configural invariance	1291.993	232	0.000	0.935	0.071	0.073							
Weak invariance	1328.713	247	0.000	0.934	0.069	0.074	Model 1 vs. model 2	36.720	15	0.001	0.001	0.002	0.001
Strong invariance	1399.335	262	0.000	0.931	0.069	0.075	Model 2 vs. model 3	70.622	15	0.000	0.003	0.000	0.001
**Academic writing – General achievement**													
Configural invariance	1331.167	348	0.000	0.936	0.071	0.072							
Weak invariance	1379.657	378	0.000	0.935	0.069	0.073	Model 1 vs. model 2	48.490	30	0.018	0.001	0.002	0.001
Strong invariance	1434.769	408	0.000	0.934	0.067	0.074	Model 2 vs. model 3	55.112	30	0.003	0.001	0.002	0.001
**Academic writing – Grade**													
Configural invariance	1775.991	696	0.000	0.938	0.070	0.067							
Weak invariance	1978.118	771	0.000	0.932	0.070	0.078	Model 1 vs. model 2	202.127	75	0.000	0.006	0.000	0.009
Strong invariance	2401.789	846	0.000	0.913	0.076	0.084	Model 2 vs. model 3	423.671	75	0.000	0.019	0.006	0.006
**Recreational writing – Gender**													
Configural invariance	817.792	232	0.000	0.916	0.080	0.066							
Weak invariance	835.075	247	0.000	0.915	0.078	0.068	Model 1 vs. model 2	17.283	15	0.302	0.001	0.002	0.002
Strong invariance	868.706	262	0.000	0.913	0.077	0.070	Model 2 vs. model 3	33.631	15	0.004	0.002	0.001	0.002
**Recreational writing – General achievement**													
Configural invariance	956.434	348	0.000	0.910	0.083	0.072							
Weak invariance	987.215	378	0.000	0.910	0.080	0.076	Model 1 vs. model 2	30.781	30	0.426	0.000	0.003	0.004
Strong invariance	1049.107	408	0.000	0.906	0.078	0.078	Model 2 vs. model 3	61.792	30	0.001	0.004	0.002	0.002
**Recreational writing – Grade**													
Configural invariance	1570.524	696	0.000	0.895	0.091	0.080							
Weak invariance	1664.695	771	0.000	0.891	0.088	0.091	Model 1 vs. model 2	94.171	75	0.066	0.004	0.003	0.010
Strong invariance	1869.357	846	0.000	0.876	0.090	0.098	Model 2 vs. model 3	204.662	75	0.000	0.015	0.002	0.007

**TABLE 7:** Multiple-group measurement invariance testing on SRQ-academic and recreational reading motivation across grade level: summary of goodness-of-fit statistics.

Measurement invariance tests	Overall results						Compared models	Model difference results					
	YBχ^2^	*df*	*p*	CFI	RMSEA	SRMR		ΔYBχ^2^	Δ*df*	*p*	ΔCFI	ΔRMSEA	ΔSRMR
**Academic reading – Middle elementary grades (Grades 3 and 4)**													
Configural invariance	477.655	232	0.000	0.943	0.053	0.058							
Weak invariance	492.196	247	0.000	0.943	0.051	0.060	Model 1 vs. model 2	14.541	15	0.485	0.000	0.002	0.002
Strong invariance	511.418	262	0.000	0.942	0.051	0.061	Model 2 vs. model 3	19.222	15	0.204	0.001	0.001	0.001
**Academic reading – Upper elementary grades (Grades 5 and 6)**													
Configural invariance	499.015	232	0.000	0.957	0.056	0.059							
Weak invariance	522.538	247	0.000	0.956	0.055	0.063	Model 1 vs. model 2	23.523	15	0.074	0.001	0.001	0.004
Strong invariance	553.289	262	0.000	0.953	0.055	0.064	Model 2 vs. model 3	30.751	15	0.009	0.003	0.000	0.001
**Academic reading – Lower secondary grades (Grades 7 and 8)**													
Configural invariance	447.332	232	0.000	0.963	0.058	0.063							
Weak invariance	465.987	247	0.000	0.963	0.056	0.065	Model 1 vs. model 2	18.655	15	0.230	0.000	0.002	0.002
Strong invariance	510.977	262	0.000	0.958	0.058	0.068	Model 2 vs. model 3	44.990	15	0.000	0.005	0.002	0.003
**Recreational reading – Middle elementary grades (Grades 3 and 4)**													
Configural invariance	623.574	232	0.000	0.926	0.070	0.068							
Weak invariance	643.977	247	0.000	0.925	0.068	0.071	Model 1 vs. model 2	20.403	15	0.157	0.001	0.002	0.003
Strong invariance	660.879	262	0.000	0.925	0.066	0.072	Model 2 vs. model 3	16.902	15	0.325	0.000	0.002	0.001
**Recreational reading – Upper elementary grades (Grades 5 and 6)**													
Configural invariance	733.053	232	0.000	0.921	0.083	0.070							
Weak invariance	753.588	247	0.000	0.921	0.080	0.072	Model 1 vs. model 2	20.535	15	0.153	0.000	0.003	0.002
Strong invariance	805.459	262	0.000	0.917	0.080	0.074	Model 2 vs. model 3	51.871	15	0.000	0.004	0.000	0.002
**Recreational reading – Lower secondary grades (Grades 7 and 8)**													
Configural invariance	467.142	232	0.000	0.967	0.064	0.062							
Weak invariance	483.916	247	0.000	0.967	0.063	0.065	Model 1 vs. model 2	16.774	15	0.333	0.000	0.001	0.003
Strong invariance	513.916	262	0.000	0.965	0.062	0.067	Model 2 vs. model 3	30.000	15	0.012	0.002	0.001	0.002

**TABLE 8:** Multiple-group measurement invariance testing on SRQ-academic and recreational writing motivation across grade level: summary of goodness-of-fit statistics.

Measurement invariance tests	Overall results						Compared models	Model difference results					
	YBχ^2^	*df*	*p*	CFI	RMSEA	SRMR		ΔYBχ^2^	Δ*df*	*p*	ΔCFI	ΔRMSEA	ΔSRMR
**Academic writing – Middle elementary grades (Grades 3 and 4)**													
Configural invariance	562.884	232	0.000	0.941	0.063	0.064							
Weak invariance	591.158	247	0.000	0.939	0.062	0.068	Model 1 vs. model 2	28.274	15	0.020	0.002	0.001	0.004
Strong invariance	621.838	262	0.000	0.936	0.062	0.069	Model 2 vs. model 3	30.680	15	0.010	0.003	0.000	0.001
**Academic writing – Upper elementary grades (Grades 5 and 6)**													
Configural invariance	596.724	232	0.000	0.938	0.070	0.067							
Weak invariance	607.608	247	0.000	0.939	0.067	0.068	Model 1 vs. model 2	10.884	15	0.761	0.001	0.003	0.001
Strong invariance	638.139	262	0.000	0.937	0.066	0.069	Model 2 vs. model 3	30.531	15	0.010	0.002	0.001	0.001
**Academic writing – Lower Secondary Grades (Grades 7 and 8)**													
Configural invariance	616.922	232	0.000	0.936	0.080	0.070							
Weak invariance	655.054	247	0.000	0.933	0.079	0.077	Model 1 vs. model 2	38.132	15	0.001	0.003	0.001	0.007
Strong invariance	714.346	262	0.000	0.927	0.080	0.080	Model 2 vs. model 3	59.292	15	0.000	0.006	0.001	0.003
**Recreational writing – Middle elementary grades (Grades 3 and 4)**													
Configural invariance	465.340	232	0.000	0.917	0.071	0.075							
Weak invariance	477.794	247	0.000	0.917	0.069	0.079	Model 1 vs. model 2	12.454	15	0.645	0.000	0.002	0.004
Strong invariance	484.377	262	0.000	0.920	0.066	0.079	Model 2 vs. model 3	6.583	15	0.968	0.003	0.003	0.000
**Recreational writing – Upper elementary grades (Grades 5 and 6)**													
Configural invariance	618.211	232	0.000	0.889	0.098	0.082							
Weak invariance	642.704	247	0.000	0.886	0.096	0.088	Model 1 vs. model 2	24.493	15	0.057	0.003	0.008	0.006
Strong invariance	681.424	262	0.000	0.880	0.096	0.091	Model 2 vs. model 3	38.720	15	0.001	0.006	0.000	0.003
**Recreational writing – Lower Secondary Grades (Grades 7 and 8)**													
Configural invariance	476.587	232	0.000	0.866	0.126	0.094							
Weak invariance	487.388	247	0.000	0.867	0.122	0.103	Model 1 vs. model 2	10.801	15	0.767	0.001	0.004	0.009
Strong invariance	507.278	262	0.000	0.865	0.120	0.105	Model 2 vs. model 3	19.890	15	0.176	0.002	0.002	0.002

### RQ2: The Relations Between the Scales

The factor correlations of the SRQ-Reading and Writing Motivation scales are presented in Table 9. These results show strong positive correlations between students’ autonomous reading and writing motivation on the one hand (i.e., correlations ranging from *r* = 0.51 to *r* = 0.94) and students’ controlled reading and writing motivation on the other hand (i.e., correlations ranging from *r* = 0.65 to *r* = 0.78). Students’ autonomous and controlled reading and writing motivation are not or slightly positively correlated with values ranging from *r* = −0.05 to *r* = 0.33. Finally, the results revealed strong correlations between students’ academic and recreational motivation (i.e., autonomous reading motivation: *r* = 0.94, *p* < 0.000, autonomous writing motivation: *r* = 0.90, *p* < 0.000, controlled reading motivation: *r* = 0.73, *p* < 0.000, and controlled writing motivation: *r* = 0.69, *p* < 0.000).

**TABLE 9:** Factor correlations reading and writing motivation.

	(1)	(2)	(3)	(4)	(5)	(6)	(7)	(8)
(1) Academic autonomous reading motivation	1							
(2) Recreational autonomous reading motivation	0.94***	1						
(3) Academic autonomous writing motivation	0.54***	0.51***	1					
(4) Recreational autonomous writing motivation	0.60***	0.61***	0.90***	1				
(5) Academic controlled reading motivation	–0.04	−0.05*	0.17***	0.10**	1			
(6) Recreational controlled reading motivation	0.19***	0.17***	0.33***	0.28***	0.73***	1		
(7) Academic controlled writing motivation	0.05	0.03	0.14***	0.08*	0.78***	0.65***	1	
(8) Recreational controlled writing motivation	0.14**	0.11**	0.32***	0.26***	0.61***	0.79***	0.69***	1

***p < 0.001, **p < 0.01, *p < 0.05.

### RQ3: Trends in Reading and Writing Motivation

**FIGURE 1:**
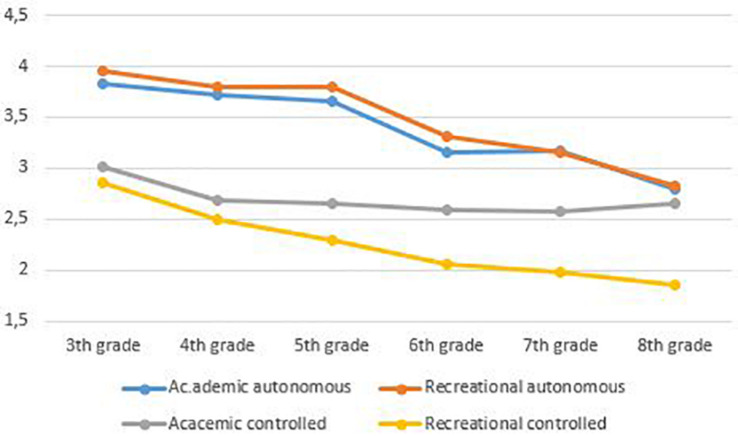
Students’ mean scores for reading motivation. Based on the results of the MG-MI (RQ1), indicating strong invariance for students within the same grade level, students’ scores within grade levels can be compared while students’ scores across grade levels cannot be compared.

**FIGURE 2:**
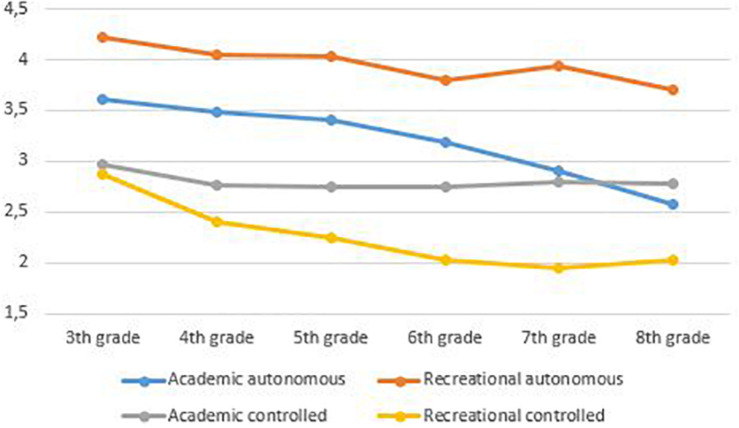
Students’ mean scores for writing motivation. Based on the results of the MG-MI (RQ1), indicating strong invariance for students within the same grade level, students’ scores within grade levels can be compared while students’ scores across grade levels cannot be compared.

Based on the results of the MG-MI, indicating strong invariance for students within the same grade level, students’ scores within grade levels and not across grade levels were compared. More specifically, multilevel analyses were conducted, testing the differences by respectively comparing: (a) third and fourth graders, (b) fifth and sixth graders, and (c) seventh and eighth graders. Table 10 presents the means and standard deviations for students’ reading and writing motivation per grade. Furthermore, significant mean differences within grade levels are presented. Finally, the overall trends in reading and writing motivation are visualized in Figures 1, 2.

**TABLE 10:** Descriptive statistics and differences within middle elementary, upper elementary, and lower secondary grades.

Mean (*SD*)				
Grade	Academic autonomous	Recreational autonomous	Academic controlled	Recreational controlled
**Reading motivation**				
3rd grade	3.83 (0.90)	3.96 (0.93)	3.01 (0.83)	2.85 (0.91)
4th grade^a^	3.72 (0.91)	3.80 (0.99)	2.69 (0.76)***	2.49 (0.82)***
5th grade	3.65 (0.88)	3.79 (0.95)	2.65 (0.76)	2.29 (0.76)
6th grade^b^	3.16 (1.06)***	3.31 (1.16)***	2.59 (0.78)	2.05 (0.70)***
7th grade	3.17 (1.05)	3.15 (1.18)	2.58 (0.71)	1.98 (0.68)
8th grade^c^	2.79 (1.06)***	2.83 (1.21)**	2.66 (0.72)	1.86 (0.66)
**Writing motivation**				
3rd grade	3.61 (1.04)	4.22 (0.73)	2.96 (0.93)	2.87 (1.05)
4th grade^a^	3.48 (1.03)	4.05 (0.73)	2.77 (0.84)**	2.41 (0.85)***
5th grade	3.40 (1.02)	4.03 (0.75)	2.75 (0.82)	2.25 (0.81)
6th grade^b^	3.19 (1.04)*	3.79 (0.94)*	2.75 (0.81)	2.03 (0.81)*
7th grade	2.90 (1.02)	3.94 (0.73)	2.80 (0.82)	1.94 (0.85)
8th grade^c^	2.57 (0.97)**	3.71 (0.96)	2.78 (0.81)	2.03 (0.94)

^a^Testing differences within middle elementary grades: 3rd grade as reference category. ^b^Testing mean differences within upper elementary grades: 5th grade as reference category. ^c^Testing mean differences within upper secondary grades: 7th grade as reference category. ***p < 0.001, **p < 0.01, *p < 0.05.

Concerning students’ autonomous reading and writing motivation, the results show that sixth graders are significantly less autonomously motivated to read and write compared to fifth graders [academic reading: χ^2^_(__1__)_ = 28.27, *p* < 0.001; recreational reading: χ^2^_(__1__)_ = 23.28, *p* < 0.001; academic writing: χ^2^_(__1__)_ = 4.93, *p* < 0.05; and recreational writing: χ^2^_(__1__)_ = 4.48, *p* < 0.05]. Likewise, eighth graders’ autonomous reading and writing motivation is significantly lower than seventh graders’ autonomous motivation [academic reading: χ^2^_(__1__)_ = 13.98, *p* < 0.001; recreational reading: χ^2^_(__1__)_ = 8.49, *p* < 0.01; and academic writing: χ^2^_(__1__)_ = 8.95, *p* < 0.01]. Regarding students’ controlled reading and writing motivation, the results show that fourth graders are significantly less controlled motivated to read and write compared to 3rd graders [academic reading: χ^2^_(__1__)_ = 15.98, *p* < 0.001; recreational reading: χ^2^_(__1__)_ = 27.79, *p* < 0.001; academic writing: χ^2^_(__1__)_ = 7.04, *p* < 0.01; and recreational writing: χ^2^_(__1__)_ = 21.08, *p* < 0.001]. Finally, when comparing sixth graders’ recreational controlled reading and writing motivation to fifth graders’ motivation, the results indicate a significant decline [recreational reading: χ^2^_(__1__)_ = 11.73, *p* < 0.001; and recreational writing: χ^2^_(__1__)_ = 4.50, *p* < 0.05].

## Discussion

The twofold aim of the present research was to substantiate the validity the SRQ-Reading and Writing Motivation scales in academic and recreational contexts in grades three to eight and to map motivational trends. The focus of the analyses was on the factor structures and correlations of the scales, its measurement invariance across gender, general achievement level, and grades and its reliability. Based on these analyses, motivational reading and writing trends were mapped in both academic and recreational contexts. In the following, we summarize and discuss the findings, address the limitations of the study, and offer directions for future research.

### The SRQ-Reading and Writing Motivation Scales

We were able to confirm the two-factor model of autonomous and controlled reading and writing motivation in both the academic and recreational context from grades three to eight using the SRQ-Reading and Writing Motivation, hereby extending prior research in the fifth and sixth grades (De Naeghel et al., 2012; De Smedt et al., 2018b). This suggests that students from grades three to eight differentiate between two qualitatively different types of motivation (i.e., autonomous and controlled motivation) and that this two-factor model is stable in academic and leisure-time reading and writing. Furthermore, the SRQ-Reading Motivation and SRQ-Writing Motivation measures both reading and writing motivation in a reliable way. Further the measurement models were invariant across gender and general achievement level. This implies that, low, average, and high achievers on the one hand and boys and girls on the other hand interpreted the SRQ-Reading and Writing items in a conceptually similar way. This further indicates that the latent factor scores and correlations of the scales can be readily compared across gender and general achievement level. However, strong invariance for the measurement models across the six different grades could not be confirmed, indicating that third, fourth, fifth, sixth, seventh, and eighth graders interpreted the SRQ-Reading Motivation and SRQ-Writing Motivation items differently. This finding partly corroborates the study on reading and writing achievement motivation of Hamilton et al. (2013). More specifically, these authors found that the reading items loaded consistently across grades (i.e., grade 2 through 7), while the interpretation of the writing items differed for younger and older students. Based on the current findings and the study of Hamilton et al. (2013), there is evidence that students at different developmental stages interpret motivational reading and/or writing items differently. This makes comparisons across grades and mapping motivational trends throughout students’ academic career difficult. Therefore, we additionally studied measurement invariance between grades within the same grade level (i.e., middle elementary, upper elementary, and lower secondary grade). Based on these results showing strong invariance for the measurement models across grades within the same grade level, we can conclude that respectively (a) third and fourth graders, (b) fifth and sixth graders, and (c) seventh and eighth graders interpreted the items of the SRQ-Reading and Writing scales similarly. Consequently, the latent factor scores and correlations can be compared across grades within the same grade level, enabling the study of motivational trends within grade levels.

### Relations Between Reading and Writing Motivation

The results confirmed the interrelatedness of reading and writing motives by revealing strong positive correlations between students’ autonomous reading and writing motivation on the one hand and students’ controlled reading and writing motivation on the other hand. In other words, the more students are motivated to read because of its perceived value or out of inherent satisfaction, the more they are motivated to write for the same reasons and vice versa. Likewise, the more students feel forced to read because of internal or external pressure, the more they write because of similar feelings of pressure. Furthermore, we found strong positive correlations between students’ academic reading and writing and their leisure-time reading and writing, which suggests being autonomously motivated to read or write for school, generally coincides with being autonomously motivated for reading or writing during their leisure time (and vice versa) as well. Similarly, students who generally feel pressured to read or write for school, are driven by similar motives for leisure-time reading or writing. Finally, the results revealed very low correlations between autonomous and controlled reading and writing motivation, indicating that autonomous and controlled motivation are distinct types of motivation that are hardly or not related within the context of academic and recreational reading and writing. These findings confirm previous research in which autonomous and controlled reading motivation (De Naeghel et al., 2012 reported correlations ranging from −0.01 to 0.07) and autonomous and controlled writing motivation (De Smedt et al., 2018b reported a correlation of 0.00) were not correlated.

By tapping into and revealing motivational reading-writing relations, the present study expands the research field on reading-writing connections, which up to now has exclusively focused on cognitive and metacognitive variables (Fitzgerald and Shanahan, 2000; Shanahan, 2016, 2019; Graham et al., 2018). Also including motivational connections in complex reading-writing models, can reveal patterns of relations between cognitive and motivational variables and may advance the literacy research field. In this respect, it is particularly worthwhile to include qualitatively different types of reading and writing motivation (i.e., autonomous and controlled motivation) in these reading-writing models since different underlying reasons to read or write can potentially be related to different cognitive and metacognitive reading and writing processes and may potentially affect students’ reading and writing performance differently (e.g., De Naeghel et al., 2012; De Smedt et al., 2018b).

### Trends in Reading and Writing Motivation

Based on the results of the measurement invariance analyses, we studied motivational trends within grade levels and not across grade levels. By mapping these motivational trends in academic and recreational reading and writing, several parallel trends between reading and writing motivation become apparent.

First, the results revealed that, in general, students’ autonomous motivation for academic and recreational reading and writing is higher than their controlled motivation. As autonomous motivation is the most optimal type of motivation (Ryan and Deci, 2000a, 2020), these results are promising: students in all grades report to read or write because of inherent satisfaction or perceived value more than because of external or internal pressure. One exception, however, can be noted: eighth graders’ controlled academic writing motivation is higher than their autonomous academic writing motivation. This suggests that these eight grade students are more driven to write for school because of feelings of internal or external pressure rather than because of its inherent satisfaction or perceived value.

Second, the results revealed an overall systematic and similar decline of both reading and writing motivation. Concerning students’ autonomous motivation, a first significant drop in academic and recreational reading and writing is situated in upper elementary grades and is followed by a second significant decline in lower secondary grades (with the exception of recreational writing). Concerning students’ controlled motivation, there is an initial decline in both academic and recreational reading and writing, which already sets in middle elementary grades. A second significant drop of students’ recreational reading and writing motivation is situated in upper elementary grades. These findings corroborate previous studies indicating that reading motivation appears to decline from the end of elementary school on (Smith et al., 2012) and that also writing motivation decreases as students progress through school (Cleary, 1991). This systematic decline in reading and writing motivation fits in with the general decline in academic motivation throughout students’ school careers as well (Gottfried et al., 2001; Bouffard et al., 2003; Corpus et al., 2009; Gnambs and Hanfstingl, 2016). Furthermore, students’ decline in reading and writing motivation also seems to coincide with the decline in Flemish students’ reading performance the past decade (Mullis et al., 2017; OECD, 2019).

Third, the present study also expands current knowledge on motivational trends by providing evidence on how the quality of students’ motives for engaging in reading and writing activities simultaneously change over time. More specifically, the motivational trends appear to develop similarly for reading and writing: regardless of the type of motivation, there is a significant decline in students’ motivation for academic and recreational reading and writing. It is especially worrying that students’ autonomous reading and writing motivation, which is the most optimal type of motivation given the positive relationships with student outcomes (De Naeghel et al., 2012; De Smedt et al., 2018b), significantly drops in upper elementary and lower secondary grades. This suggests that students, as they progress through school, are less and less driven by autonomous motives to engage in literacy activities both in and beyond school. In other words, they are less driven by their inherent love for reading and writing or by their awareness of its importance. This finding confirms previous studies on general academic motivation indicating that students exhibit a marked decline in academic intrinsic motivation that already sets in at an early age (Gottfried et al., 2001; Bouffard et al., 2003; Corpus et al., 2009; Gnambs and Hanfstingl, 2016). More specifically, Gottfried et al. (2001) reported that academic intrinsic motivation starts to decline from the age of 9 and continues to decline to the age of 16. The decline of students’ controlled reading and writing motivation already sets in from middle elementary grades through upper elementary grades: students feel less and less forced to read or write because of internal or external pressure. The overall decline of both autonomous and controlled motivation is worrying because this trend seems to indicate that nothing really moves students to read or write, indicating students are moving toward amotivation.

Taking into account the abovementioned trends in students’ reading and writing motivation, we can conclude that the end of elementary grades and the beginning of secondary grades are crucial phases for students to engage in autonomously motivating literacy activities since their autonomous motivation to read and write, both in and beyond school, seriously drops. Based on previous research on students’ declining academic intrinsic motivation, different reasons for this decline can be put forward (Gnambs and Hanfstingl, 2016). Next to developmental changes, such as identity formation (Faye and Sharpe, 2008), and neuropsychological changes, such as students’ still developing brain structures (Blakemore et al., 2007), SDT-related research puts forward a need-driven explanation (Gnambs and Hanfstingl, 2016). More specifically, SDT points to the importance of fostering autonomous motivation by nurturing students’ inherent psychological need for autonomy (i.e., feeling psychologically free), competence (i.e., feeling confident and effective), and relatedness (i.e., feeling related to significant others) (Ryan and Deci, 2000b, 2020). In this respect, the longitudinal cohort study of Gnambs and Hanfstingl (2016) demonstrated that students’ intrinsic motivation remains fairly stable during adolescence when students experience an adequate satisfaction of these three basic psychological needs in school. To ensure the facilitation of these needs, teachers can adopt a qualitatively supportive teaching style, characterized by autonomy-supportive, structured, and involved teacher behavior (Soenens and Vansteenkiste, 2005). In the context of reading and writing instruction, some experimental studies aiming at fostering students’ autonomous reading or writing motivation already exist (e.g., De Naeghel et al., 2016; De Smedt et al., 2018a, 2020), but remain rather scarce. Therefore, more research is needed to identify and test instructional reading and writing activities that promote students’ autonomous motivation. Given the interrelatedness between both literacy activities, teachers can nurture students’ need for autonomy, competence, and relatedness in several ways. For instance, teachers can (a) provide a large collection of books from which students can choose and provide a wide range of writing tasks wherein they can, for example, write a summary of what they read or write a specific book recommendation (i.e., autonomy), (b) instruct, model, and guide students to effectively apply reading and writing strategies when writing an end to a story they read (i.e., competence), or (c) enable the publication of texts written by students so peers can read them or give the floor to writers who can read their story out loud in front of the class (i.e., relatedness). The effectiveness of such instructional reading and writing activities should be evaluated in future research studies in terms of the effect on both motivational and cognitive outcomes in reading and writing.

### Limitations and Suggestions for Future Research

In addition to the recommendations put forward in discussing the results above, we conclude with acknowledging some limitations and presenting suggestions for future research. First, we were unable to confirm strong invariance for the measurement models of the SRQ Reading and Writing Motivation across the six different grades. This implies that third, fourth, fifth, sixth, seventh, and eighth graders interpreted the SRQ-Reading and Writing Motivation items differently. Future research should invest in obtaining more fine-grained insights into these interpretation differences by means of cognitive pretesting (i.e., asking students to explain or paraphrase the questionnaire’s items and explain their responses) (Woolley et al., 2006) and by developing different grade-level appropriate measures of reading and writing motivation. Additionally, future research is encouraged to test the predictive and incremental validity of the SRQ-Reading Motivation and SRQ-Writing Motivation scales.

Second, we collected cross-sectional data to study trends in students’ motivation to read and write. Based on these cross-sectional data it is, however, impossible to reflect upon developmental changes in students’ motivation since age and cohort are confounded (Miyazaki and Raudenbush, 2000). To map developmental trends in students’ motivation, longitudinal study designs are needed. Depending on the overall research aim and the available resources, there are several options for longitudinal designs which future research studies can adopt. First, future studies can use a two-cohort longitudinal design in which students enroll in third grade (cohort 1) or fifth grade (cohort 2) and participate for 4 years (e.g., Hamilton et al., 2013). Using this design, students’ reading and writing motivation can be tracked over time and developmental changes can be assessed and mapped. However, if future studies want to control better for possible cohort effects, a multiple cohort in accelerated longitudinal designs might be worthwhile to consider (Miyazaki and Raudenbush, 2000). Following this design, multiple age cohorts should be sampled and longitudinal data on members of each cohort are to be collected so age effects can be disentangled from cohort effects. It is, however, important to note that these longitudinal studies should adopt grade-level appropriate measures for reading and writing motivation. In both the two-cohort and multiple cohort design, including a cohort of lower elementary grade students, who were not included in the current study, could be considered as well (e.g., Stutz et al., 2017). Including this group of beginning readers and writers would broaden the overall picture by providing insights into students’ initial reading and writing motivation.

Third, future studies should additionally include students’ reading and writing performance when analyzing trends in reading and writing motivation. Indeed, it is possible that students with poor reading and writing skills have different motives for reading and writing compared to higher achieving readers and writers. Additionally, future research should study whether poor readers and writers show a greater decline in their reading and writing motivation over time. Moreover, including reading and writing tests in view of assessing students’ reading and writing performance is a more objective measure for student achievement than the more subjective teacher estimations for academic achievement as was used in the present study.

Fourth, the decline in students’ motivation for reading and writing is internationally acknowledged (Mullis et al., 2017; OECD, 2019). Although the current study has been conducted in Flanders (Belgium), future research should study how students’ reading and writing motivation evolves throughout their school career from an international perspective. In this respect, comparative research should investigate if and how national educational contexts might foster or hamper students’ reading and writing motivation. To do so, reliable and valid instruments based on an unequivocal theoretical framework, are needed to measure students’ reading and writing motivation across gender, achievement level, and grades.

Finally, given the lack of studies on motivational reading-writing models, future studies should shed light on causal reading-writing relations (e.g., how can initial reading motivation in lower elementary grades affect writing motivation in middle elementary grades). In this respect, future studies can rely on latent variable panel analyses of longitudinal data to investigate patterns of direct and indirect relations among reading and writing motivation over time (Little et al., 2007).

### Implications

The SRQ-Reading and Writing Motivation scales applied in the present study are valuable for researchers interested in assessing middle elementary, upper elementary, and lower secondary students’ autonomous and controlled reading and writing motivation in both academic and recreational contexts. The multi-dimensionality of the scales, highlighting qualitatively different types of reading and writing motivation, makes these scales sensitive for different motives which incites students to read or write for school or during their free time. Based on previous research, we know that there is a positive relation between autonomous motivation and reading and writing outcomes on the one hand and a negative relation between controlled motivation and reading and writing performance on the other hand (De Naeghel et al., 2012; De Smedt et al., 2018b). Consequently, the SRQ-Reading and Writing Motivation scales are valid and reliable scales to study not only the quantity, but also the quality of students’ reading and writing motivation. Furthermore, these scales can provide more nuanced insights into the impact of specific instructional reading and writing interventions on students’ reading and writing motivation (e.g., De Smedt et al., 2018a). As such, we can gain a deeper and more nuanced understanding of how we can foster the most optimal motives for reading and writing throughout students’ academic careers.

## Data Availability Statement

The datasets are available on request to the corresponding author.

## Ethics Statement

The studies involving human participants were reviewed and approved by the Faculty of Psychology and Educational Sciences of Ghent University (General Ethical Protocol for Scientific Research). Written informed consent for participation was not provided by the participants’ legal guardians/next of kin because: the data was collected in 2016 before GDPR regulations were in effect. There was an active written informed consent from the participants’ school principal and a passive written informed consent from participants’ parents.

## Author Contributions

FD, AR, and HV designed the study. FD and AR were in charge of the data collection procedure. SH and FD analyzed the data. All the authors wrote and reviewed the manuscript and approved its final version.

## Conflict of Interest

The authors declare that the research was conducted in the absence of any commercial or financial relationships that could be construed as a potential conflict of interest.
