# Supplementary material for: Assessing and Mapping Reading and Writing Motivation in Third to Eight Graders: A Self-Determination Theory Perspective
Source: Front Psychol. 2020 Jul 28;11:1678. doi: 10.3389/fpsyg.2020.01678 (PMC7399692; doi:10.3389/fpsyg.2020.01678)
Supplement: Supplementary file 1 [file Table_1.DOCX]

Supplementary Material

# Supplementary Table 1

Confirmatory Factor Analyses on the SRQ-Reading and Writing Motivation: Summary of Goodness-of-Fit Statistics per Grade Level

| **Academic Reading Motivation** | | | | | |
| --- | --- | --- | --- | --- | --- |
|  | YBχ² | *df* | CFI | RMSEA | SRMR |
| Middle elementary grades (*n* = 885) | 318.416*** | 116 | .951 | .049 | .051 |
| Upper elementary grades (*n* = 843) | 367.124*** | 116 | .961 | .054 | .055 |
| Lower secondary grades (*n* = 615) | 342.713*** | 116 | .961 | .056 | .060 |
| All grades (*n* = 2343) | 772.715*** | 116 | .956 | .055 | .058 |
| **Recreational Reading Motivation** | | | | | |
|  | YBχ² | *df* | CFI | RMSEA | SRMR |
| Middle elementary grades (*n* = 885) | 485.573*** | 116 | .930 | .068 | .064 |
| Upper elementary grades (*n* = 843) | 614.565*** | 116 | .924 | .082 | .068 |
| Lower secondary grades (*n* = 615) | 323.794*** | 116 | .970 | .062 | .055 |
| All grades (*n* = 2343) | 1127.497*** | 116 | .943 | .073 | .064 |
| **Academic Writing Motivation** | | | | | |
|  | YBχ² | *df* | CFI | RMSEA | SRMR |
| Middle elementary grades (*n* = 885) | 465.969*** | 116 | .937 | .065 | .060 |
| Upper elementary grades (*n* = 843) | 481.894*** | 116 | .937 | .070 | .061 |
| Lower secondary grades (*n* = 615) | 501.688*** | 116 | .934 | .081 | .073 |
| All grades (*n* = 2343) | 1147.748*** | 116 | .937 | .071 | .071 |
| **Recreational Writing Motivation** | | | | | |
|  | YBχ² | *df* | CFI | RMSEA | SRMR |
| Middle elementary grades (*n* = 885) | 390.342*** | 116 | .905 | .078 | .069 |
| Upper elementary grades (*n* = 843) | 396.432*** | 116 | .910 | .087 | .078 |
| Lower secondary grades (*n* = 615) | 261.111*** | 116 | .906 | .103 | .074 |
| All grades (*n* = 2343) | 687.707*** | 116 | .918 | .080 | .064 |
