# Supplementary material for: Assessing and Mapping Reading and Writing Motivation in Third to Eight Graders: A Self-Determination Theory Perspective
Source: Front Psychol. 2020 Jul 28;11:1678. doi: 10.3389/fpsyg.2020.01678 (PMC7399692; doi:10.3389/fpsyg.2020.01678)
Supplement: Supplementary file 2 [file Table_2.DOCX]

Supplementary Material

# Supplementary Table 2

SRQ-Reading Motivation: Items and Standardized Factor Loadings for Academic Reading per Grade Level

| Item | Autonomous | | | | | Controlled | | | *R²* | | |
| --- | --- | --- | --- | --- | --- | --- | --- | --- | --- | --- | --- |
| **I read for school because…** | A^a^ | | B^b^ | | C^c^ | A | B | C | A | B | C |
| I **enjoy** reading. | .74 | | .79 | | .78 |  | | | .55 | .62 | .61 |
| I think it is **very useful** for me to read. | .69 | .77 | | .82 | |  | | | .48 | .59 | .68 |
| It’s **fun** to read. | .80 | .86 | | .88 | |  | | | .65 | .74 | .77 |
| I **really like it**. | .77 | .86 | | .90 | |  | | | .59 | .74 | .81 |
| I think reading is **meaningful**. | .65 | .76 | | .85 | |  | | | .42 | .57 | .73 |
| I think reading is **interesting**. | .77 | .85 | | .90 | |  | | | .59 | .73 | .81 |
| It is **important to me to read**. | .60 | .72 | | .85 | |  | | | .36 | .51 | .71 |
| I think reading is **fascinating**. | .36 | .77 | | .89 | |  | | | .13 | .59 | .79 |
| I don’t want to **disappoint others**. |  | | | | | .59 | .63 | .61 | .35 | .39 | .37 |
| That is what **others expect me to do**. |  | | | | | .57 | .63 | .66 | .32 | .40 | .44 |
| I will feel **guilty** if I don’t do it. |  | | | | | .66 | .67 | .70 | .44 | .45 | .59 |
| **Others will only reward me if I read**. |  | | | | | .58 | .58 | .59 | .33 | .34 | .35 |
| I have t**o prove to myself that I can get good reading grades**. |  | | | | | .35 | .44 | .40 | .12 | .20 | .16 |
| **Others will punish me** if I don’t read. |  | | | | | .62 | .63 | .55 | .38 | .40 | .30 |
| I will feel **ashamed** of myself if I don’t red. |  | | | | | .68 | .68 | .68 | .46 | .47 | .46 |
| **Others think that I have to**. |  | | | | | .64 | .68 | .61 | .41 | .46 | .37 |
| I can just be **proud of myself if I get good reading grades**. |  | | | | | .34 | .38 | .36 | .12 | .14 | .13 |
| *Note.* ^a^ Middle elementary grades  ^b^ Upper elementary grades  ^c^ Lower secondary grades | | | | | | | | | | | |
