# Supplementary material for: Assessing and Mapping Reading and Writing Motivation in Third to Eight Graders: A Self-Determination Theory Perspective
Source: Front Psychol. 2020 Jul 28;11:1678. doi: 10.3389/fpsyg.2020.01678 (PMC7399692; doi:10.3389/fpsyg.2020.01678)
Supplement: Supplementary file 3 [file Table_3.DOCX]

Supplementary Material

# Supplementary Table 3

SRQ-Reading Motivation: Items and Standardized Factor Loadings for Recreational Reading per Grade Level

| Item | Autonomous | | | | | Controlled | | | *R²* | | |
| --- | --- | --- | --- | --- | --- | --- | --- | --- | --- | --- | --- |
| **I read in my free time because…** | A^a^ | | B^b^ | | C^c^ | A | B | C | A | B | C |
| I **enjoy** reading. | .81 | | .84 | | .89 |  | | | .66 | .71 | .80 |
| I think it is **very useful** for me to read. | .76 | .76 | | .88 | |  | | | .58 | .58 | .78 |
| It’s **fun** to read. | .82 | .88 | | .92 | |  | | | .68 | .76 | .85 |
| I **really like it**. | .82 | .86 | | .92 | |  | | | .67 | .74 | .85 |
| I think reading is **meaningful**. | .74 | .80 | | .91 | |  | | | .55 | .64 | .82 |
| I think reading is **interesting**. | .81 | .88 | | .93 | |  | | | .66 | .78 | .87 |
| It is **important to me to read**. | .61 | .76 | | .88 | |  | | | .37 | .58 | .77 |
| I think reading is **fascinating**. | .41 | .79 | | .91 | |  | | | .17 | .62 | .82 |
| I don’t want to **disappoint others**. |  | | | | | .72 | .70 | .76 | .51 | .50 | .57 |
| That is what **others expect me to do**. |  | | | | | .72 | .70 | .73 | .52 | .50 | .52 |
| I will feel **guilty** if I don’t do it. |  | | | | | .69 | .73 | .73 | .47 | .53 | .54 |
| **Others will only reward me if I read**. |  | | | | | .64 | .62 | .71 | .41 | .39 | .50 |
| I have t**o prove to myself that I can get good reading grades**. |  | | | | | .41 | .43 | .45 | .16 | .19 | .20 |
| **Others will punish me** if I don’t read. |  | | | | | .57 | .61 | .62 | .33 | .37 | .39 |
| I will feel **ashamed** of myself if I don’t red. |  | | | | | .69 | .68 | .73 | .47 | .46 | .54 |
| **Others think that I have to**. |  | | | | | .68 | .66 | .70 | .46 | .43 | .48 |
| I can just be **proud of myself if I get good reading grades**. |  | | | | | .36 | .38 | .50 | .13 | .15 | .25 |
| *Note.* ^a^ Middle elementary grades  ^b^ Upper elementary grades  ^c^ Lower secondary grades | | | | | | | | | | | |
