# Supplementary material for: Assessing and Mapping Reading and Writing Motivation in Third to Eight Graders: A Self-Determination Theory Perspective
Source: Front Psychol. 2020 Jul 28;11:1678. doi: 10.3389/fpsyg.2020.01678 (PMC7399692; doi:10.3389/fpsyg.2020.01678)
Supplement: Supplementary file 4 [file Table_4.DOCX]

Supplementary Material

# Supplementary Table 4

SRQ-Writing Motivation: Items and Standardized Factor Loadings for Academic Writing per Grade Level

| Item | Autonomous | | | | | Controlled | | | *R²* | | |
| --- | --- | --- | --- | --- | --- | --- | --- | --- | --- | --- | --- |
| **I write a text for school because…** | A^a^ | | B^b^ | | C^c^ | A | B | C | A | B | C |
| I **enjoy** writing. | .77 | | .79 | | .80 |  | | | .59 | .63 | .64 |
| I think it is **very useful** for me to write. | .72 | .70 | | .81 | |  | | | .51 | .50 | .65 |
| It’s **fun** to write. | .81 | .82 | | .86 | |  | | | .66 | .68 | .74 |
| I **really like it**. | .79 | .84 | | .87 | |  | | | .62 | .70 | .75 |
| I think writing is **meaningful**. | .75 | .77 | | .84 | |  | | | .57 | .59 | .70 |
| I think writing is **interesting**. | .80 | .86 | | .90 | |  | | | .64 | .75 | .80 |
| It is **important to me to write**. | .70 | .71 | | .83 | |  | | | .48 | .51 | .69 |
| I think writing is **fascinating**. | .47 | .81 | | .88 | |  | | | .22 | .65 | .77 |
| I don’t want to **disappoint others**. |  | | | | | .72 | .71 | .71 | .52 | .50 | .50 |
| That is what **others expect me to do**. |  | | | | | .73 | .63 | .70 | .54 | .39 | .50 |
| I will feel **guilty** if I don’t do it. |  | | | | | .70 | .75 | .75 | .49 | .56 | .56 |
| **Others will only reward me if I write**. |  | | | | | .60 | .58 | .57 | .36 | .33 | .32 |
| I have t**o prove to myself that I can get good writing grades**. |  | | | | | .34 | .43 | .49 | .11 | .18 | .24 |
| **Others will punish me** if I don’t write. |  | | | | | .60 | .63 | .58 | .36 | .39 | .34 |
| I will feel **ashamed** of myself if I don’t write. |  | | | | | .70 | .69 | .71 | .49 | .47 | .51 |
| **Others think that I have to**. |  | | | | | .73 | .68 | .73 | .53 | .46 | .53 |
| I can just be **proud of myself if I get good writing grades**. |  | | | | | .33 | .37 | .50 | .11 | .14 | .25 |
| *Note.* ^a^ Middle elementary grades  ^b^ Upper elementary grades  ^c^ Lower secondary grades | | | | | | | | | | | |
