# Supplementary material for: Assessing and Mapping Reading and Writing Motivation in Third to Eight Graders: A Self-Determination Theory Perspective
Source: Front Psychol. 2020 Jul 28;11:1678. doi: 10.3389/fpsyg.2020.01678 (PMC7399692; doi:10.3389/fpsyg.2020.01678)
Supplement: Supplementary file 5 [file Table_5.DOCX]

Supplementary Material

# Supplementary Table 5

SRQ-Writing Motivation: Items and Standardized Factor Loadings for Recreational Writing per Grade Level

| Item | Autonomous | | | | | Controlled | | | *R²* | | |
| --- | --- | --- | --- | --- | --- | --- | --- | --- | --- | --- | --- |
| **I write a text in my free time because…** | A^a^ | | B^b^ | | C^c^ | A | B | C | A | B | C |
| I **enjoy** writing. | .80 | | .80 | | .78 |  | | | .63 | .65 | .61 |
| I think it is **very useful** for me to write. | .59 | .71 | | .71 | |  | | | .35 | .50 | .50 |
| It’s **fun** to write. | .82 | .83 | | .82 | |  | | | .67 | .69 | .67 |
| I **really like it**. | .80 | .78 | | .84 | |  | | | .64 | .61 | .71 |
| I think writing is **meaningful**. | .52 | .74 | | .78 | |  | | | .27 | .55 | .61 |
| I think writing is **interesting**. | .58 | .81 | | .92 | |  | | | .46 | .65 | .84 |
| It is **important to me to write**. | .50 | .69 | | .80 | |  | | | .25 | .47 | .64 |
| I think writing is **fascinating**. | .26 | .69 | | .84 | |  | | | .07 | .47 | .70 |
| I don’t want to **disappoint others**. |  | | | | | .77 | .73 | .80 | .60 | .53 | .63 |
| That is what **others expect me to do**. |  | | | | | .75 | .79 | .89 | .57 | .63 | .79 |
| I will feel **guilty** if I don’t do it. |  | | | | | .78 | .72 | .77 | .60 | .52 | .60 |
| **Others will only reward me if I write**. |  | | | | | .68 | .66 | .87 | .47 | .44 | .76 |
| I have t**o prove to myself that I can get good writing grades**. |  | | | | | .39 | .38 | .57 | .15 | .15 | .32 |
| **Others will punish me** if I don’t write. |  | | | | | .65 | .64 | .71 | .42 | .40 | .50 |
| I will feel **ashamed** of myself if I don’t write. |  | | | | | .77 | .70 | .75 | .59 | .49 | .56 |
| **Others think that I have to**. |  | | | | | .76 | .72 | .79 | .58 | .52 | .63 |
| I can just be **proud of myself if I get good writing grades**. |  | | | | | .39 | .42 | .61 | .15 | .18 | .37 |
| *Note.* ^a^ Middle elementary grades  ^b^ Upper elementary grades  ^c^ Lower secondary grades | | | | | | | | | | | |
