# Supplementary material for: Assessing and Mapping Reading and Writing Motivation in Third to Eight Graders: A Self-Determination Theory Perspective
Source: Front Psychol. 2020 Jul 28;11:1678. doi: 10.3389/fpsyg.2020.01678 (PMC7399692; doi:10.3389/fpsyg.2020.01678)
Supplement: Supplementary file 6 [file Table_6.DOCX]

Supplementary Material

# Supplementary Table 6

Reliability measures: SRQ-Reading and Writing Motivation per Grade Level (Bentler’s *ρ*)

|  | Middle elementary grades | Upper elementary grades | Lower secondary grades |
| --- | --- | --- | --- |
| Academic reading motivation |  |  |  |
| Autonomous | .86 | .93 | .96 |
| Controlled | .80 | .81 | .80 |
| Recreational reading motivation |  |  |  |
| Autonomous | .89 | .94 | .97 |
| Controlled | .83 | .80 | .81 |
| Academic writing motivation |  |  |  |
| Autonomous | .89 | .93 | .95 |
| Controlled | .83 | .82 | .84 |
| Recreational writing motivation |  |  |  |
| Autonomous | .81 | .91 | .94 |
| Controlled | .86 | .80 | .89 |
